# Exclusive image guided IMRT vs. radical prostatectomy followed by postoperative IMRT for localized prostate cancer: a matched-pair analysis based on risk-groups

**DOI:** 10.1186/1748-717X-7-158

**Published:** 2012-09-17

**Authors:** Caroline Azelie, Mélanie Gauthier, Céline Mirjolet, Luc Cormier, Etienne Martin, Karine Peignaux-Casasnovas, Gilles Truc, Jérôme Chamois, Philippe Maingon, Gilles Créhange

**Affiliations:** 1 Department of Radiation Oncology, Anticancer center Georges François, Leclerc, 1 rue du Professeur Marion, 21000, Dijon, France; 2 Department of Biostatistics, Anticancer center Georges François, Leclerc, 1 rue du Professeur Marion, 21000, Dijon, France; 3 Department of Urology, University Hospital Le Bocage, Bd de Lattre de Tassigny, 21000, Dijon, France; 4 Department of Oncological Surgery, Anticancer center Georges François, Leclerc, 1 rue du Professeur Marion, 21000, Dijon, France

**Keywords:** Prostate cancer, IGRT, IMRT, Postoperative radiotherapy, Exclusive radiotherapy

## Abstract

**Background:**

To investigate whether patients treated for a localized prostate cancer (PCa) require a radical prostatectomy followed by postoperative radiotherapy or exclusive radiotherapy, in the modern era of image guided IMRT.

**Methods:**

178 patients with PCa were referred for daily exclusive image guided IMRT (IG-IMRT) using an on-line 3D ultra-sound based system and 69 patients were referred for postoperative IMRT without image guidance after radical prostatectomy (RP + IMRT). Patients were matched in a 1:1 ratio according to their baseline risk group before any treatment. Late toxicity was scored using the CTV v3.0 scale. Biochemical failure was defined as a postoperative PSA ≤ 0.1 ng/mL followed by 1 consecutive rising PSA for the postoperative group of patients and by the Phoenix definition (nadir + 2 ng/mL) for the group of patients treated with exclusive radiotherapy.

**Results:**

A total of 98 patients were matched (49:49). From the start of any treatment, the median follow-up was 56.6 months (CI 95% = [49.6-61.2], range [18.2-115.1]). No patient had late gastrointestinal grade ≥ 2 toxicity in the IG-IMRT group vs. 4% in the RP + IMRT group. Forty two percent of the patients in both groups had late grade ≥ 2 genitourinary toxicity. The 5-year FFF rates in the IG-IMRT group and in the RP + IMRT groups were 93.1% [80.0-97.8] and 76.5% [58.3-87.5], respectively (p = 0.031).

**Conclusions:**

Patients with a localized PCa treated with IG-IMRT had better oncological outcome than patients treated with RP + IMRT. Further improvements in postoperative IMRT using image guidance and dose escalation are urgently needed.

## Background

Prostate cancer (PCa) is one of the leading causes of death among men in Europe and the United States [1,2]. For localized PCa, several therapeutic options can be proposed to the patients in a curative intent, and as a result there is considerable controversy about whether conservative treatments give better results than non-conservative treatments. At diagnosis, the selection of patients for treatment is based on clinical characteristics (i.e. age, results from digital rectal examination, PSA value and TRUS-based sextant-biopsy), which may lead to either under or over-treatment.

With radical prostatectomy (RP) alone, 30% to 50% of the PCa patients will suffer from biochemical failure [3-5]. Improved staging methods including preoperative MRI to predict the risk of extracapsular extension, seminal vesicle involvement or positive margin and advances and improvements in surgical techniques and procedures have contributed to the use of RP in intermediate- or high-risk patients [5,6]. In the case of high-risk features revealed in the examination of pathological specimens assessment, adjuvant radiotherapy (aRT) is a standard approach, and has led to acceptable and encouraging rates of 5-year biochemical-relapse free survival ranging between 72% and 74% [7,8]. Even salvage radiotherapy (sRT) for biochemical failures, when delivered early after biochemical failure (i.e. PSA < 0.5 ng/mL), gives acceptable rates of 5-year biochemical-relapse free survival [9]. In the context of a risk-adapted multimodal approach, 10-year cancer-specific survival rates that can be reached range between 88% and 92%, so that it is now widely accepted that this strategy including postoperative radiotherapy with or without androgen deprivation therapy (ADT) is one of the standards of care for patients with high-risk disease [8,10].

With an adequate dose of radiation (≥ 72 Gy), exclusive radiotherapy seems to be comparable to RP in terms of biochemical control [11-14]. Nevertheless, although several randomized trials have confirmed that patients with PCa treated with exclusive 3D conformal radiotherapy benefit from dose escalation, late toxicity have also significantly increased in parallel [15-17].

Technological advances such as IGRT and IMRT have been developed, thereby allowing dose escalation to the prostate with reduced toxicity and maintained biochemical control [18,19]. The use of combined IMRT and IGRT (IG-IMRT) is rapidly growing, when the prostate has not been removed. Very low rates of toxicity with high rates of 5-year biochemical control have been reported with IG-IMRT [18,20].

Although there is considerable controversy about the best treatment for localized prostate cancer, whatever the risk group, the advent of IMRT and IGRT worldwide prompted us to carry out a pragmatic study to compare the impact of RP followed by postoperative IMRT with exclusive IG-IMRT in a matched-pair analysis based on risk-groups as determined preoperatively.

## Methods

### Patients

Between 01/2002 and 12/2009, 178 with localized PCa were referred for exclusive radiotherapy (IG-IMRT). In the same period and following RP, 69 patients were referred for aRT or early sRT without image guidance (RP + IMRT).

All of the patients underwent a prior bone scan and pelvic CT scan to rule out nodal and/or distant disease. Patients with a risk of nodal involvement at diagnosis had to undergo pelvic lymph node dissection for nodal staging if their risk was 15% or higher. As MRI and MR spectroscopy are now recognized ways to improve PCa detection and mapping and staging, all of the patients underwent a baseline MRI with combined MR spectroscopy at 3 Tesla to detect extra-capsular extension or seminal vesicle invasion, both of which could preclude for surgery, thus improving T-staging and the selection of high-risk patients before treatment. Pretherapeutic trans-rectal ultra-sound-based sextant biopsies (≥ 6 cores) with a validated and standardized procedure were also performed. Patients were matched one to one according to the baseline risk group as defined by D’Amico’s classification assessed before exclusive radiotherapy or surgery.

After approval from the Institutional Review Board of the Georges François Leclerc Cancer Center, a total of 98 patients were matched (49:49).

### Postoperative IMRT (n = 49)

Patient repositioning was performed daily based on skin-marks alignment. Thereafter, two orthogonal 2D images were acquired to use bony landmarks as the standard reference for repositioning before treatment with on-board electronic portal imaging (ePID). These were matched with related Beam’s Eye Views as determined on the 3D planning CT through days 1 to 3 to correct for set-up uncertainties, and then checked weekly until completion of radiotherapy.

### Exclusive IG-IMRT (n = 49)

Daily image guidance was performed using a 3D US-based system (Son Array®, Varian Medical Systems). The technique for US-based image guidance used in our institution has been described in detail elsewhere [21].

### Follow-up

Follow-up evaluations after treatment were performed at intervals of 3 to 6 months for 5 years and then annually. Each evaluation included digital rectal examination, PSA value and an evaluation of toxicity. Late toxicity was scored retrospectively according to the Common Toxicity Criteria Adverse Events version 3 (CTCAE v.3.0) morbidity grading scale.

### Statistics

To evaluate the efficacy of the two radiotherapy scheme (exclusive IG-IMRT vs. RP + postoperative IMRT), patients were matched one to one according to their baseline risk group (as defined using the classification of D'Amico) before any treatment. Differences between subgroups were calculated using a Chi-square or an exact Fischer test for categorical data and using Student’s test or Kruskall Wallis test for continuous data.

In the RP + IMRT group, two definitions of a biochemical failure were tested : a PSA ≥ 0.1 ng/mL followed by a consecutive rising PSA value and a PSA ≥ 0.2 ng/mL followed by a consecutive rising PSA value. In the exclusive IG-IMRT group, biochemical failure was defined by the Phoenix definition (nadir of PSA + 2 ng/mL). The rate of freedom from failure (FFF) was defined as the time between the first day of any treatment and the date of biochemical failure using the Kaplan Meier method. Patients were censored at last follow-up or death without relapse. The log-rank statistic was used to test for differences between groups. Univariate analysis was used to determine the predictive factors for time to biochemical relapse (using Cox proportional hazard ratio model) and for late toxicities (using Logistic Regression).

P values were two-sided and considered significant when lesser than 0.05. All of the analyses were performed using STATA V11 software (STATA Corp, College Station, TX).

## Results

Unsurprisingly, patients in the RP + IMRT group were more likely to be younger than patients selected for exclusive IG-IMRT (p < 0.001). At baseline, the matched patients were more likely to have a low- or intermediate-risk disease. The characteristics of the patients, the tumors and treatment with respect to the treatment arm are summarized in Table 1.

**Table 1:** Patient and tumor characteristics for IG-IMRT (n = 49) and RP followed by IMRT (n = 49)

**Characteristics**	**Total**	**RP + IMRT**	**IG-IMRT**	**p-value**
	**(n = 98)**	**(n = 49)**	**(n = 49)**	
Median age – years [range]	66.8 [51.7-82.7]	64.8 [51.7-82.7]	70.2 [53.3-82.4]	< 0.001 ^£^
Median follow-up – months [range]	56.6 [18.22-115.1]	51.5 [18.2-115.1]	62.2 [25.5-106.0]	
Clinical T stage				1.0 ^$^
T1c – n (%)	60 (61.2%)	30 (61.2%)	30 (61.2%)	
T2 – n (%)	4 (4.1%)	2 (4.1%)	2 (4.1%)	
T2a – n (%)	14 (14.3%)	7 (14.3%)	7 (14.3%)	
T2b – n (%)	20 (20.4%)	10 (20.4%)	10 (20.4%)	
Gleason score on biopsy				1.0 ^μ^
≤ 6 – n (%)	58 (59.2%)	29 (59.2%)	29 (59.2%)	
7 – n (%)	40 (40.8%)	20 (40.8%)	20 (40.8%)	
Pretherapeutic PSA				0.632 ^#^
Median value – ng/mL [range]	8.7 [1.7 - 32]	8.7 [2.7 - 29]	8.7 [1.7 - 32]	
Risk group				1.0 ^$^
Low	30 (30.6%)	15 (30.6%)	15 (30.6%)	
Intermediate	62 (63.3%)	31 (63.3%)	31 (63.3%)	
High	6 (6.1%)	3 (6.1%)	3 (6.1%)	
Concomitant hormones with radiation				1.0 ^μ^
n (%)	20 (20.4%)	10 (20.4%)	10 (20.4%)	
Adjuvant hormones with radiation				0.564 ^μ^
n (%)	14 (14.3%)	6 (12.2%)	8 (16.3%)	
Mean dose of radiotherapy				
Prostatic fossa – median value (Gy) [range]	70.0 [31.4 – 79.8]	66.0 [31.4 – 70.3]	77.4 [73.0 – 79.8]	< 0.001 ^#^
Rectum – median value (Gy) [range]	38.1 [4.8 – 61.1]	36.0 [4.8 – 57.1]	39.3 [26.5 – 61.1]	0.061 ^#^
Bladder – median value (Gy) [range]	32.7 [15.5 – 67.3]	43.6 [16.8 – 63.7]	31.3 [15.5 – 67.3]	< 0.001 ^£^

* Union for International Cancer Control classification 2002 (6^th^ edition); £ Student’s test; $ Fisher’s exact test; # Kruskall Wallis test; μ: Chi2.

*IG-IMRT*: Image-guided intensity modulated radiotherapy; *RP*: radical prostatectomy, *PSA*: Prostate Specific Antigen.

### Surgery and postoperative IMRT (n = 49)

For the group of patients who underwent a RP (n = 49), the pathological stage was pT2b for 3 patients (6.1%), pT2c for 18 patients (36.7%), pT3a for 16 patients (32.6%) and pT3b for 12 patients (24.5%). Therefore, after comparisons with pretherapeutic clinical stage as defined using digital rectal examination, and sextant-biopsy, all of the tumors were upstaged after pathological evaluation of the specimen. Pelvic lymph node dissection was performed in 40 patients (81.6%) of whom none had nodal involvement according to the pathology examination. A microscopic positive surgical margin was observed in 30 patients (65.2%). The Gleason score evaluated on specimen was ≤ 6 in 19 patients (38.7%), 7 in 29 patients (59.2%) and ≥ 8 in 1 patient (2.0%). Comparisons between the pre and postoperative Gleason score showed that the Gleason score was upgraded after the pathological examination of the specimen in 17 patients (34.5%). Postoperative PSA was detectable for 22 patients (45.8%).

The median delay between surgery and postoperative IMRT was 11.4 months [2.9-69.6]. Seventeen patients (35%) underwent aRT whereas 32 patients (65%) had early sRT. The median PSA value before postoperative IMRT was 0.28 ng/mL [0.00 – 6.99]. Post-operative IMRT delivered daily fractions of 2 Gy, (5 fractions a week) using the same protocol for all the patients.

### Late toxicity (n = 96)

The median dose delivered in the IG-IMRT group was 77.4 Gy [73.0-79.8]. In the postoperative setting, the median radiation dose was 66.0 Gy [31.4-70.3 Gy].

We found no difference between the group of patients treated with exclusive IG-IMRT and the group of patients treated with RP followed by postoperative IMRT for gastrointestinal toxicity or genitourinary toxicity. The rates of late grade ≥ 2 gastrointestinal toxicity at 5 years were 0% (0/49)in the IG-IMRT group and 4% (2/47) in the RP + IMRT group, whereas the rates of late grade ≥ 2 genitourinary toxicity were 42% (21/49) and 42% (20/47) respectively (p = 0.976). Late grade 2 or higher toxicities are summarized in Table 2.

**Table 2:** Late toxicity for localized prostate cancer patients with respect to treatment arm (IG-IMRT vs. RP followed by IMRT)

**Toxicity**	**Total**	**IG-IMRT**	**RP + IMRT**	**p-value**
	**(n= 96)**	**(n= 49)**	**(n= 47)**	
Genitourinary				0.145
G0	30 (31.3%)	11 (22.5%)	19 (40.4%)	
G1	25 (26.0%)	17 (34.7%)	8 (17.0%)	
G2	24 (25.0%)	12 (24.5%)	12 (25.5%)	
G3	17 (17.7%)	9 (18.4%)	8 (17.0%)	
G4	-	-	-	
Gastrointestinal				0.095
G0	85 (88.5%)	42 (85.7%)	43 (91.5%)	
G1	9 (9.4%)	7 (14.3%)	2 (4.3%)	
G2	-	-	-	
G3	2 (2.1%)	-	2 (4.3%)	
G4		-	-	

### Freedom from biochemical failure

In the entire cohort of patients (n = 98), 12 patients had failed biochemically (3 patients in the IG-IMRT group and 9 patients in the RP + IMRT group). The median Kaplan Meier reverse follow-up for the entire cohort was 56.6 months (CI95%: [49.6-61.2]); range: (18.22-115.1), 51.5 months in the RP + IMRT group (CI95%: [39.2-59.5%]; range (18.2-115.1) months) and 62.2 months in the IG-IMRT group (CI95%: [52.3-69.5]; range (25.5-106.0) months).

The 3-year and 5-year FFF rates for the entire cohort were 91.7% [84.0%-95.7%] and 85.6% [75.7%-91.7%]. In the IG-IMRT group, the 3-year and 5-year FFF rates were 95.9% [84.5%-98.9%] and 93.1% [80.0-97.8], respectively. In the group of radical prostatectomy, the FFF rates were significantly lower when using either a PSA threshold of ≥ 0.1 ng/mL : 87.6% [74.5%-94.2%] at 3-years and 76.5% [58.3-87.5] at 5-years (p = 0.031) or with a PSA threshold of ≥ 0.2 ng/mL : 87.5% [74.3%-94.2%] at 3-years and 75.2% [55.7-87.0] at 5-years (p = 0.032).

**Figure 1:**
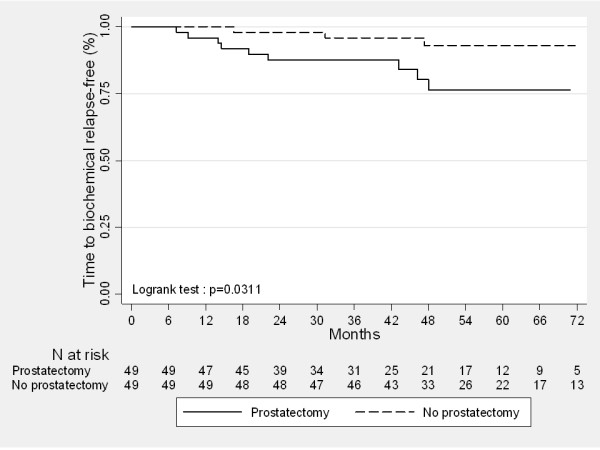
Kaplan Meier curves comparing biochemical relapse-free survival for localized prostate cancer patients treated with exclusive IG-IMRT and radical prostatectomy followed by IMRT calculated from the start of any treatment (p = 0.031, univariate analysis).

Time to biochemical relapse-free curves according to the treatment sequence is represented in Figure 1. In univariate analysis, RP followed by postoperative IMRT was the only predictive factor of an increased biochemical failure rate after treatment (Table 3).

**Table 3:** Three and 5-year FFF rates from the start of any treatment with comparison of patient-, tumor- and treatment-related characteristics (univariate analysis)

**Characteristics**	**Events (n)/patients (n)**	**3-year FFF rates - % [range]**	**5-year FFF rates - % [range]**	**Log-rank test**
				**p-value**
Treatment				0.0311*
IG-IMRT	3/49	95.87% [84.49%-98.95%]	93.14% [79.96%-97.76%]	
RP + IMRT	9/49	87.61% [74.48%-94.24%]	76.46% [58.31%-87.50%]	
Age				
≤ 66.8 years	5/49	91.74% [79.47%-96.82%]	88.35% [73.58%-95.12%]	0.595
> 66.8 years	7/49	91.59% [79.08%-96.76%]	83.00% [67.17%-91.65%]	
Tumor stage				
T1	8/60	91.44% [80.61%-96.35%]	84.37% [70.75%-91.99%]	0.758
T2	4/38	92.11% [77.49%-97.38%]	87.72% [69.57%-95.38%]	
Gleason score				
≤ 6	5/58	96.52% [86.80%-99.12%]	89.15% [75.57%-95.40%]	0.135
7	7/40	84.68% [68.98%-92.82%]	81.29% [64.45%-90.69%]	
Pretherapeutic PSA				
≤ 8.7 ng/mL	4/49	93.83% [82.08%-97.97%]	90.99% [77.46%-96.57%]	0.250
> 8.7 ng/mL	8/49	89.55% [76.67%-95.52%]	80.69% [64.58%-90.01%]	
Concomitant ADT				
No	12/78	89.53% [80.13%-94.63%]	82.02% [70.12%-89.52%]	0.070
Yes	0/20	100 [ND – ND]	100 [ND – ND]	
Adjuvant ADT				
No	12/84	90.29% [81.51%-95.03%]	83.44% [72.36%-90.36%]	0.156
Yes	0/14	100 [ND – ND]	100 [ND – ND]	

*FFF*: Freedom from biochemical failure; *ADT*: androgen deprivation therapy; * the definition of biochemical failure in the RP + IMRT group is a *PSA* threshold of ≥ 0.1 ng/mL.

## Discussion

In the absence of a randomized trial directly comparing RP with treatment by radiation therapy in men with localized PCa, it is often difficult for patients and physicians to determine which treatment option to pursue for localized PCa.

The results from several larger retrospective trials indicate that biochemical and survival outcomes with RP, dose-escalated external beam radiotherapy and brachytherapy are similar [13,14,22]. In a large trial that compared RP with exclusive radiotherapy in 1682 men with PCa, the strongest predictive factors of a biochemical relapse were the pretherapeutic PSA value, the Gleason score on biopsy, the clinical T-stage and the radiation dose for patients who underwent radiation therapy [11]. Previous studies in which the dose of radiotherapy delivered to the prostate was suboptimal (< 72 Gy) showed worse outcomes with external radiotherapy than with RP for low- or intermediate-risk PCa patients [11,13,14].

Dose escalation up to > 74 Gy benefits all risk-groups of patients in terms of biochemical relapse-free survival [16,23]. With a longer follow-up, a positive impact of dose escalation on survival has been also observed [24]. Intensity modulated radiation therapy to deliver dose-escalated radiotherapy gives promising biochemical outcomes with a dramatic decrease in rectal toxicity. It is therefore widely accepted as safe for PCa patients selected for exclusive radiotherapy [25].

When the prostate has not been removed, IGRT with or without IMRT, has also demonstrated similar outcomes with very low rates of late grade ≥ 3 gastrointestinal and genitourinary toxicity in less than 5% of the patients, respectively [18].

In-parallel, improvements in surgery and pretherapeutic imaging have been tested so that even intermediate or high-risk patients at baseline could also benefit from RP. Nevertheless, approximately 15-25% of men who undergo RP for localized prostate cancer will experience biochemical failure (i.e. men with features of more severe disease in the pathological evaluation of the specimen, such as extension through the capsule, seminal vesicle involvement, a positive surgical margin and/or a a higher Gleason score). Adjuvant postoperative radiotherapy in this setting improves biochemical control [6,26]. Some authors even advocate that early salvage radiotherapy with a careful monitoring of PSA postoperatively provides similar results, thereby limiting the adverse urinary effects of postoperative radiotherapy [9].

Previous clinical studies have attempted to compare biochemical outcome in RP and exclusive radiotherapy in patients with a clinically localized PCa [11,12,14,22]. Although larger than our study, all of the previously published retrospective comparisons of RP and exclusive radiotherapy for localized PCa suffer from a heterogeneous design with no distinction between patients who did or did not require adjuvant radiation. In the absence of a randomized trial, we chose to perform a matched-pair analysis by selecting patients who required postoperative radiotherapy after pathologically proven poor characteristics. By using this methodology, we found that patients treated with exclusive IG-IMRT had better 5-year FFF than those treated with RP followed by postoperative IMRT. Despite the strengths of our study, such as the matched-pair comparison on risk-groups as defined clinically at baseline, our study suffers from some limitations. Major limitations arise from the retrospective design, with drawbacks related to selection bias and sample size. Some could argue that the likelihood of pathologically unexpected high-risk features is unknown in the IG-IMRT group. Nevertheless, we believe that the findings of this pragmatic study will be helpful in the modern era of IMRT and IGRT, in that they suggest how the results of RP can be improved, as the choice of treatment always depends on baseline clinical characteristics. Moreover, multiparametric MRI is part of the work-up procedure in our institution. Multiparametric MRI has dramatically improved the detection of T3 [27]. We therefore assumed that the patients in the IG-IMRT group closely matched those in the RP + IMRT group. Although we found that IMRT, given with combined IGRT or postoperatively without image guidance, resulted in very low rates of toxicity in both groups, our results suggest that image guidance could play a major role in local control. The incremental value of IGRT combined with exclusive IMRT suggests that IG-IMRT should also be tested in the postoperative setting. With the advent of on-board CT imaging such as Cone Beam Computed Tomography (CBCT), postop IGRT based on soft tissues (i.e. the prostate bed) gives more accurate radiation delivery in the postop setting than does 2D images of the bony landmarks. Preliminary experience with RP followed by postoperative IGRT using 3D CBCT for repositioning based on soft tissues has already been reported with promising findings compared with 2D images [28]. It could also be expected that results from postoperative radiotherapy could be improved by escalating the dose of radiotherapy. A dose effect has also been clearly demonstrated in this setting with a significant impact on biochemical control for doses above 72 Gy [29,30].

## Conclusions

Patients with localized PCa at diagnosis who were treated with IG-IMRT or RP + IMRT had low rates of late gastrointestinal and genitourinary toxicity. Nevertheless, patients treated with high dose IG-IMRT had significantly a better 5-year FFF, suggesting that the results of postoperative IMRT could be improved by implementing IGRT technique and/or increasing the dose of radiation above 72 Gy.

## Abbreviations

PCa, Prostate cancer; PSA, Prostate Specific Antigen; TRUS, Trans-rectal ultrasound; RP, Radical prostatectomy; MRI, Magnetic resonance imaging; aRT, Adjuvant radiotherapy; sRT, Salvage radiotherapy; ADT, Androgen deprivation therapy; IG-IMRT, Image-guided IMRT; IMRT, Intensity Modulated Radiation Therapy; IGRT, Image Guided Radiation Therapy; ePID, Electronic Portal Imaging; CTCAE v3.0, Common Toxicity Criteria Adverse Events version 3.0; FFF, Freedom From Failure; CBCT, Cone Beam computed tomography.

## Competing interests

The authors declare that they have no competing interests.

## Authors’ contributions

Conception and design: CA, CM, LC, PM, GC. Acquisition of Data: CA, EM, KP, GT, JC. Analysis and interpretation of data: MG, CA, CM, LC, PM, GC. Drafting the manuscript or revising it critically for important intellectual content: CA, CM, MG, PM, GC. All authors read and approved the final manuscript.

## References

[B1] Jemal A Bray F Center MM Ferlay J Ward E Forman D Global cancer statistics CA Cancer J Clin 2011 61 69 90 10.3322/caac.20107 21296855

[B2] Gronberg H Prostate cancer epidemiology Lancet 2003 361 859 864 10.1016/S0140-6736(03)12713-4 12642065

[B3] Han M Partin AW Zahurak M Piantadosi S Epstein JI Walsh PC Biochemical (prostate specific antigen) recurrence probability following radical prostatectomy for clinically localized prostate cancer J Urol 2003 169 517 523 10.1016/S0022-5347(05)63946-8 12544300

[B4] Porter CR Kodama K Gibbons RP Correa R Jr Chun FK Perrotte P Karakiewicz PI 25-year prostate cancer control and survival outcomes: a 40-year radical prostatectomy single institution series J Urol 2006 176 569 574 10.1016/j.juro.2006.03.094 16813891

[B5] Roehl KA Han M Ramos CG Antenor JA Catalona WJ Cancer progression and survival rates following anatomical radical retropubic prostatectomy in 3,478 consecutive patients: long-term results J Urol 2004 172 910 914 10.1097/01.ju.0000134888.22332.bb 15310996

[B6] Ward JF Slezak JM Blute ML Bergstralh EJ Zincke H Radical prostatectomy for clinically advanced (cT3) prostate cancer since the advent of prostate-specific antigen testing: 15-year outcome BJU Int 2005 95 751 756 10.1111/j.1464-410X.2005.05394.x 15794776

[B7] Bolla M van Poppel H Collette L van Cangh P Vekemans K Da Pozzo L de Reijke TM Verbaeys A Bosset JF van Velthoven R Postoperative radiotherapy after radical prostatectomy: a randomised controlled trial (EORTC trial 22911) Lancet 2005 366 572 578 10.1016/S0140-6736(05)67101-2 16099293

[B8] Thompson IM Tangen CM Paradelo J Lucia MS Miller G Troyer D Messing E Forman J Chin J Swanson G Adjuvant radiotherapy for pathological T3N0M0 prostate cancer significantly reduces risk of metastases and improves survival: long-term followup of a randomized clinical trial J Urol 2009 181 956 962 10.1016/j.juro.2008.11.032 19167731 PMC3510761

[B9] Stephenson AJ Scardino PT Kattan MW Pisansky TM Slawin KM Klein EA Anscher MS Michalski JM Sandler HM Lin DW Predicting the outcome of salvage radiation therapy for recurrent prostate cancer after radical prostatectomy J Clin Oncol 2007 25 2035 2041 10.1200/JCO.2006.08.9607 17513807 PMC2670394

[B10] Boorjian SA Karnes RJ Crispen PL Rangel LJ Bergstralh EJ Blute ML Radiation therapy after radical prostatectomy: impact on metastasis and survival J Urol 2009 182 2708 2714 10.1016/j.juro.2009.08.027 19836762

[B11] Kupelian PA Elshaikh M Reddy CA Zippe C Klein EA Comparison of the efficacy of local therapies for localized prostate cancer in the prostate-specific antigen era: a large single-institution experience with radical prostatectomy and external-beam radiotherapy J Clin Oncol 2002 20 3376 3385 10.1200/JCO.2002.01.150 12177097

[B12] Martinez AA Gonzalez JA Chung AK Kestin LL Balasubramaniam M Diokno AC Ziaja EL Brabbins DS Vicini FA A comparison of external beam radiation therapy versus radical prostatectomy for patients with low risk prostate carcinoma diagnosed, staged, and treated at a single institution Cancer 2000 88 425 432 10.1002/(SICI)1097-0142(20000115)88:2<425::AID-CNCR25>3.0.CO;2-Z 10640977

[B13] D'Amico AV Whittington R Malkowicz SB Cote K Loffredo M Schultz D Chen MH Tomaszewski JE Renshaw AA Wein A Richie JP Biochemical outcome after radical prostatectomy or external beam radiation therapy for patients with clinically localized prostate carcinoma in the prostate specific antigen era Cancer 2002 95 281 286 10.1002/cncr.10657 12124827

[B14] Kupelian PA Potters L Khuntia D Ciezki JP Reddy CA Reuther AM Carlson TP Klein EA Radical prostatectomy, external beam radiotherapy <72 Gy, external beam radiotherapy > or =72 Gy, permanent seed implantation, or combined seeds/external beam radiotherapy for stage T1-T2 prostate cancer Int J Radiat Oncol Biol Phys 2004 58 25 33 10.1016/S0360-3016(03)00784-3 14697417

[B15] Huang EH Pollack A Levy L Starkschall G Dong L Rosen I Kuban DA Late rectal toxicity: dose-volume effects of conformal radiotherapy for prostate cancer Int J Radiat Oncol Biol Phys 2002 54 1314 1321 10.1016/S0360-3016(02)03742-2 12459352

[B16] Peeters ST Heemsbergen WD van Putten WL Slot A Tabak H Mens JW Lebesque JV Koper PC Acute and late complications after radiotherapy for prostate cancer: results of a multicenter randomized trial comparing 68 Gy to 78 Gy Int J Radiat Oncol Biol Phys 2005 61 1019 1034 10.1016/j.ijrobp.2004.07.715 15752881

[B17] Storey MR Pollack A Zagars G Smith L Antolak J Rosen I Complications from radiotherapy dose escalation in prostate cancer: preliminary results of a randomized trial Int J Radiat Oncol Biol Phys 2000 48 635 642 10.1016/S0360-3016(00)00700-8 11020558

[B18] De Meerleer GO Fonteyne VH Vakaet L Villeirs GM Denoyette L Verbaeys A Lummen N De Neve WJ Intensity-modulated radiation therapy for prostate cancer: late morbidity and results on biochemical control Radiother Oncol 2007 82 160 166 10.1016/j.radonc.2006.12.007 17222931

[B19] Zelefsky MJ Chan H Hunt M Yamada Y Shippy AM Amols H Long-term outcome of high dose intensity modulated radiation therapy for patients with clinically localized prostate cancer J Urol 2006 176 1415 1419 10.1016/j.juro.2006.06.002 16952647

[B20] Crehange G Mirjolet C Gauthier M Martin E Truc G Peignaux-Casasnovas K Azelie C Bonnetain F Naudy S Maingon P Clinical impact of margin reduction on late toxicity and short-term biochemical control for patients treated with daily on-line image guided IMRT for prostate cancer Radiother Oncol 2012 103 2 244 246 10.1016/j.radonc.2011.10.025 22119374

[B21] Peignaux K Truc G Blanchard N Crehange G Maingon P Stage I endometrial carcinoma Cancer Radiother 2008 12 625 629 10.1016/j.canrad.2008.07.004 18706845

[B22] Tewari A Raman JD Chang P Rao S Divine G Menon M Long-term survival probability in men with clinically localized prostate cancer treated either conservatively or with definitive treatment (radiotherapy or radical prostatectomy) Urology 2006 68 1268 1274 10.1016/j.urology.2006.08.1059 17169648

[B23] Dearnaley DP Sydes MR Graham JD Aird EG Bottomley D Cowan RA Huddart RA Jose CC Matthews JH Millar J Escalated-dose versus standard-dose conformal radiotherapy in prostate cancer: first results from the MRC RT01 randomised controlled trial Lancet Oncol 2007 8 475 487 10.1016/S1470-2045(07)70143-2 17482880

[B24] Valicenti R Lu J Pilepich M Asbell S Grignon D Survival advantage from higher-dose radiation therapy for clinically localized prostate cancer treated on the radiation therapy oncology group trials J Clin Oncol 2000 18 2740 2746 10894874 10.1200/JCO.2000.18.14.2740

[B25] Alicikus ZA Yamada Y Zhang Z Pei X Hunt M Kollmeier M Cox B Zelefsky MJ Ten-year outcomes of high-dose, intensity-modulated radiotherapy for localized prostate cancer Cancer 2011 117 1429 1437 10.1002/cncr.25467 21425143

[B26] Van Poppel H Joniau S An analysis of radical prostatectomy in advanced stage and high-grade prostate cancer Eur Urol 2008 53 253 259 10.1016/j.eururo.2007.10.009 17949893

[B27] Seitz M Shukla-Dave A Bjartell A Touijer K Sciarra A Bastian PJ Stief C Hricak H Graser A Functional magnetic resonance imaging in prostate cancer Eur Urol 2009 55 801 814 10.1016/j.eururo.2009.01.027 19185981

[B28] Showalter TN Nawaz AO Xiao Y Galvin JM Valicenti RK A cone beam CT-based study for clinical target definition using pelvic anatomy during postprostatectomy radiotherapy Int J Radiat Oncol Biol Phys 2008 70 431 436 10.1016/j.ijrobp.2007.06.026 17869021

[B29] Ost P Fonteyne V Villeirs G Lumen N Oosterlinck W De Meerleer G Adjuvant high-dose intensity-modulated radiotherapy after radical prostatectomy for prostate cancer: clinical results in 104 patients Eur Urol 2009 56 669 675 10.1016/j.eururo.2009.05.041 19501453

[B30] Ost P Lumen N Goessaert AS Fonteyne V De Troyer B Jacobs F De Meerleer G High-dose salvage intensity-modulated radiotherapy with or without androgen deprivation after radical prostatectomy for rising or persisting prostate-specific antigen: 5-year results Eur Urol 2011 60 842 849 10.1016/j.eururo.2011.04.021 21514039

